# 基于生物信息学分析探究维奈克拉对急性髓系白血病的治疗敏感性及耐药机制

**DOI:** 10.3760/cma.j.cn121090-20250114-00028

**Published:** 2025-05

**Authors:** 阳 杨, 成华 许, 宁 王, 金婷 范, 丹丹 杨, 明明 牛, 龙 申, 洪 王

**Affiliations:** 1 天津医科大学，细胞生态海河实验室，天津 300070 Haihe Laboratory of Cell Ecosystem, Tianjin Medical University, Tianjin 300070, China; 2 中国医学科学院血液病医院（中国医学科学院血液学研究所），血液与健康全国重点实验室，国家血液系统疾病临床医学研究中心，细胞生态海河实验室，天津 300020 State Key Laboratory of Experimental Hematology, National Clinical Research Center for Blood Diseases, Haihe Laboratory of Cell Ecosystem, Institute of Hematology & Blood Diseases Hospital, Chinese Academy of Medical Sciences & Peking Union Medical College, Tianjin 300020, China; 3 天津医学健康研究院，天津 301600 Tianjin Institutes of Health Science, Tianjin 301600, China

**Keywords:** 维奈克拉, 急性髓系白血病, 生信分析, 耐药, Venetoclax, Acute myeloid leukemia, Bioinformatics analysis, Drug resistance

## Abstract

**目的:**

探究维奈克拉对急性髓系白血病（AML）的抑癌作用及耐药机制。

**方法:**

从Beat AML数据库下载BCL-2抑制剂维奈克拉药敏试验的AML患者基因组、转录组及临床信息数据，对其与药敏试验结果进行相关性分析。通过对转录组数据进行差异分析，筛选出与维奈克拉治疗敏感性相关的差异基因并使用GEO数据库转录组结果及体外实验进行验证。同时应用KEGG、GSEA以及KnockTF转录因子富集分析及公共数据库进一步探讨影响药物敏感性的关键基因。

**结果:**

过滤得到52例进行维奈克拉药敏试验的患者数据。分析结果表明，携带FLT3突变的患者相比无FLT3突变的患者对维奈克拉表现出更高的敏感性。临床信息与药敏试验结果的相关性分析结果显示，外周血肿瘤负荷较高的患者对维奈克拉更敏感。转录组数据分析及体外实验验证维奈克拉抑制FLT3相关信号通路。KEGG、KnockTF转录因子富集分析提示维奈克拉耐药与FOXM1、STAT3转录活性升高有关，高表达FOXM1、STAT3的患者生存期更短。

**结论:**

维奈克拉可以抑制FLT3相关信号通路激活，而STAT3和FOXM1转录因子的激活可能是维奈克拉耐药的关键机制之一。

## Abstract (en)

**Objective:**

To investigate the anti-leukemic effects and resistance mechanisms of venetoclax in acute myeloid leukemia（AML）. Genomic, transcriptomic, and clinical data from AML patients who underwent venetoclax drug sensitivity testing were downloaded from the Beat AML database. Correlation analysis was performed between these data and venetoclax sensitivity outcomes. Differentially expressed genes（DEGs）associated with venetoclax sensitivity were identified from transcriptomic data and subsequently validated using GEO database transcriptomic results and in vitro experiments（including Western blot）. Functional enrichment analyses（KEGG and GSEA）, transcription factor enrichment analysis（KnockTF）, and data from public databases were employed to further investigate key genes and pathways influencing drug sensitivity.

**Results:**

After filtering the Beat AML cohort, data from 52 patient samples with available in vitro venetoclax sensitivity results were included for analysis. Patients with FLT3 mutations exhibited greater sensitivity to venetoclax compared to those with FLT3 wild-type. Correlation analysis between clinical information and drug sensitivity data indicated that higher peripheral blood tumor burden was associated with increased sensitivity to venetoclax. Transcriptomic analysis and in vitro experiments confirmed that venetoclax inhibits the FLT3-related signaling pathway, including downregulation of FLT3 expression and reduced phosphorylation of its downstream targets AKT and STAT5. KEGG pathway and KnockTF transcription factor enrichment analyses indicated that venetoclax resistance was associated with increased transcriptional activity of FOXM1 and STAT3. Moreover, high expression of FOXM1 and STAT3 correlated with shorter overall survival in patients.

**Conclusion:**

Venetoclax can inhibit the activation of FLT3-related signaling pathways. The activation of STAT3 and FOXM1 transcription factors is a potential key mechanism contributing to venetoclax resistance in AML.

急性髓系白血病（Acute myeloid leukemia, AML）是一种以骨髓、血液和其他组织中原始和幼稚髓系细胞异常浸润为主要特征的恶性血液系统疾病，具有高度异质性[1]。当前AML的标准治疗方案是阿糖胞苷与蒽环类药物联用的“3+7”方案以及造血干细胞移植[2]，但其治愈率仍然处于较低水平，老年患者5年生存率小于20％[3]。

近年来FDA陆续批准了多种用于AML治疗的靶向药，一定程度上提高了缓解率并延长了患者的生存期[4]。研究显示，BCL-2的抑制剂维奈克拉联合阿扎胞苷治疗AML患者可以显著提高老年患者生存率[5]，且携带ASXL1或RUNX1突变的患者对治疗更敏感[6]–[7]。然而耐药性问题，包括其他抗凋亡蛋白（MCL-1、BCL-XL）表达增多、TP53突变及线粒体代谢重编程等，限制了疗效的持续性[8]。揭示潜在的精准诊疗靶标及耐药机制，是当前针对靶向BCL-2治疗AML研究的重点。本研究基于Beat AML数据库，结合基因组、转录组信息，筛选调控维奈克拉对AML治疗敏感性的关键基因及耐药靶点，现报道如下。

## 材料与方法

一、数据来源

从Beat AML数据库（http://www.vizome.org/）获取52例接受维奈克拉体外药敏试验的AML患者全外显子组、转录组及临床资料。纳入标准为具有体外药敏试验及转录组数据的样本。排除标准为符合以下任意一条：①无骨髓或外周血肿瘤细胞比例；②无总生存期信息；③无血常规或血生化信息。

二、生物信息学分析

在Beat AML数据库中在线生成维奈克拉药物敏感性与基因突变关联的火山图，以效应值[9]评估药物敏感性。同时收集52例患者的体外药敏试验数据，采用剂量-反应曲线下面积（AUC）[10]量化药物敏感性，并对其临床信息进行相关性分析。

根据AUC值，将患者分为敏感组（前25％，AUC<62）和耐药组（后25％，AUC>220）。提取转录组数据中编码蛋白的基因进行质控和差异表达分析，以校正后的*P*<0.05和|差异倍数（Fold change）|≥4作为筛选标准。采用基因集富集分析（GSEA）解析富集的信号通路，并通过标准化富集评分（NES）评估通路基因的富集情况，以|标准化富集得分（NES）|>1和校正后的*P*<0.05代表显著富集的判定标准。

针对转录组富集到的转录调控相关信号通路，利用KnockTF数据库（https://bio.liclab.net/KnockTF/index.php）对差异表达基因进行转录因子富集分析。并结合Beat AML数据库分析转录因子表达水平与维奈克拉AUC值的关系，以AUC平均值作为分组阈值，评估转录因子在药物敏感性中的作用。

为了进一步验证研究发现，检索GEO数据库中的相关转录组数据集（GSE269245、GSE199159），分析目标基因在独立数据集中的表达情况，以确认研究结果的可靠性。

三、细胞培养及试剂

AML细胞株OCI-AML2购买于南京科佰公司，使用含10％胎牛血清的MEM培养基培养。p-FLT3（#60413）、p-AKT（#9271）、p-ERK 1/2（#9101）、FLT3（#3462）、AKT（#9272）、ERK 1/2（#9102）抗体和内参GAPDH（#5174）购买于美国Cell Signaling公司。p-STAT5A（#D155020）、STAT5A（#D220085）和二抗（#D110069）购买自生工生物工程（上海）公司。二硝基亚砜（DMSO，#D8371）购买于北京索莱宝科技有限公司，维奈克拉（#SJ-MX0031）购买自山东思科捷生物技术有限公司，按照1 mg溶于1.1515 ml DMSO溶液，配制为1 mmol/L储存液。

四、Western blot检测FLT3相关信号通路蛋白表达情况

收集DMSO或维奈克拉（20 nmol/L）处理OCI-AML2细胞48 h细胞后的沉淀，300×*g*离心5 min，并使用预冷PBS洗涤两次。随后加入含有蛋白酶抑制剂的RIPA细胞裂解液提取总蛋白，并使用Pierce™ BCA Protein Assay Kit试剂盒（美国Thermo Fisher公司）定量，将蛋白浓度调整为2 µg/µl，加入1×SDS后于100 °C加热10 min使蛋白变性。

在预制胶中每孔加入20 µg蛋白样本，在80 V恒压下进行SDS-PAGE电泳2 h。电泳后，按照凝胶、PVDF膜、滤纸及海绵的顺序组装转膜体系，置于预冷转膜液中，在90 V恒压转膜2 h。转膜后用5％脱脂牛奶室温封闭1 h，随后分别使用p-FLT3（1∶1000）、p-AKT（1∶1000）、p-ERK 1/2（1∶1000）、p-STAT5（1∶1000）、FLT3（1∶1000）、AKT（1∶1000）、ERK 1/2（1∶1000）、STAT5（1∶1000）、GAPDH（1∶1000）一抗4 °C孵育过夜。次日使用TBST清洗3次，每次10 min，加入相应二抗（1∶5000）室温孵育1 h，再用TBST清洗3次，每次10 min。最后利用ECL化学发光试剂盒进行显色并用化学发光成像仪分析，评估不同药物处理条件下的蛋白表达情况。

五、统计学处理

使用GraphPad Prism 9及R 4.2.2软件进行数据分析。使用Glass's delta进行效应值估计，采用独立样本*t*检验进行组间比较，利用Spearman相关系数计算临床信息之间的相关性，以*P*<0.05为差异有统计学意义。多基因生物信息学分析中采用FDR对*P*值进行校正。使用corrplot 0.95、ggplot2 3.5.1和ggpubr 0.6.0包对临床数据进行相关性分析及可视化展示，limma 3.62.1筛选差异表达基因，ComplexHeatmap 2.14.0绘制差异基因热图，并利用clusterProfiler 4.14.4进行通路富集分析。此外，利用Kaplan-Meier Plotter数据库（https://kmplot.com/analysis/index.php?p=home）、R包survival 3.8-3及survminer 0.3.0绘制生存曲线，评估基因表达对AML预后的影响。

## 结果

一、维奈克拉治疗的AML患者基因组数据分析

如图1A所示，FLT3、SF3B1、KRAS等基因突变与维奈克拉治疗敏感性相关。在52例AML患者中，FLT3、NPM1、NRAS及DNMT3A是体细胞突变频率较高的基因（图1B）。其中，FLT3突变（FLT3-ITD或TKD突变）患者的AUC显著低于非突变患者（*P*＝0.033）（图1C），提示FLT3突变可能与维奈克拉治疗的高敏感性相关。

**图1:**
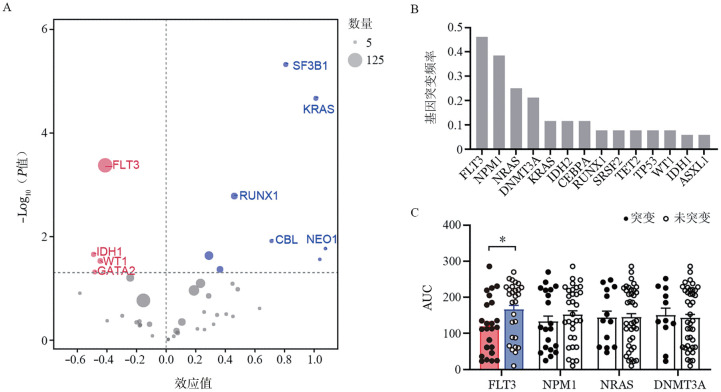
维奈克拉药物反应性与急性髓系白血病（AML）患者基因型的相关性分析（*P<0.05） **A** 不同基因突变对维奈克拉的效应值，红色代表与致敏相关，蓝色代表与耐药相关；**B** AML患者体细胞突变频率统计；**C** 不同AML体细胞突变亚组维奈克拉剂量-反应曲线下面积（AUC）比较

二、AML患者对维奈克拉临床治疗敏感性分析

在52例AML患者中，AUC与外周血肿瘤细胞比例、未成熟粒细胞比例、单核细胞比例及中性粒细胞比例具有线性相关性（均*P*<0.05）（图2）。外周血肿瘤细胞比例较高的AML患者对维奈克拉更敏感（*r*＝−0.31，*P*＝0.027），外周血未成熟粒细胞比例、单核细胞比例及中性粒细胞比例低的AML患者对维奈克拉的敏感性更高（*r*分别为0.31、0.64、0.39，均*P*<0.05），提示不同外周血细胞成分可能影响AML患者对维奈克拉的治疗反应。

**图2:**
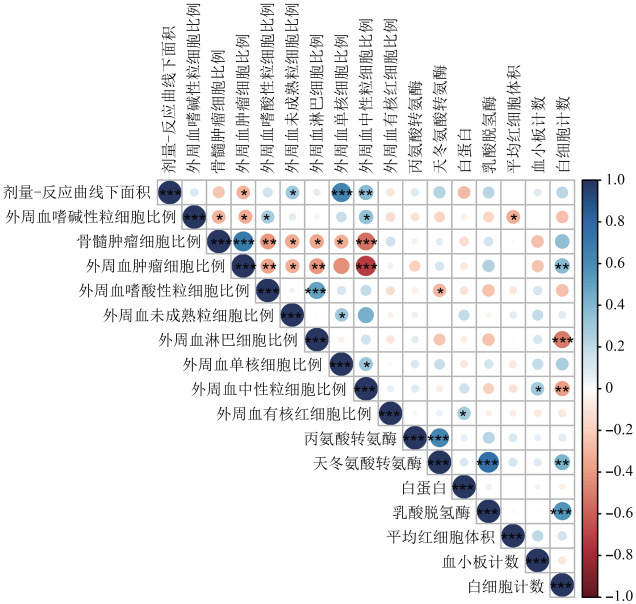
维奈克拉剂量-反应曲线下面积与急性髓系白血病患者临床指标的Spearman相关性热图（*P<0.05、**P<0.01、^***^P<0.001）

三、差异表达基因的筛选

对维奈克拉耐药组和敏感组进行差异分析，筛选出739个差异表达基因，其中耐药组相对于敏感组有554个表达上调基因，185个下调基因（图3A）。下调基因的KEGG通路分析结果显示，PI3K-AKT信号通路、花生四烯酸代谢以及蛋白质代谢等通路与AML对维奈克拉的敏感性高度相关（图3B）。此外，如图3C所示，敏感组的FLT3表达水平显著高于耐药组（*P*＝0.016）。采用GSEA分析发现，FLT3基因集在本转录组数据中显著富集（NES＝−1.445，校正后*P*＝0.020）（图3D）。为了进一步验证上述发现，检索GSE199159数据集，结果显示在MOLM13细胞中，维奈克拉处理组的FLT3表达水平显著低于对照（DMSO）组（*P*＝0.002）（图3E），同时KEGG分析结果显示，维奈克拉能够抑制FLT3相关信号通路激活（图3F）。进一步的体外实验表明，维奈克拉处理OCI-AML2细胞48 h后，FLT3、AKT、STAT5总蛋白及其磷酸化水平显著低于对照（DMSO）组（图3G）。这些结果表明，维奈克拉能够抑制FLT3及其相关信号通路的激活，从而影响AML细胞的药物敏感性。

**图3:**
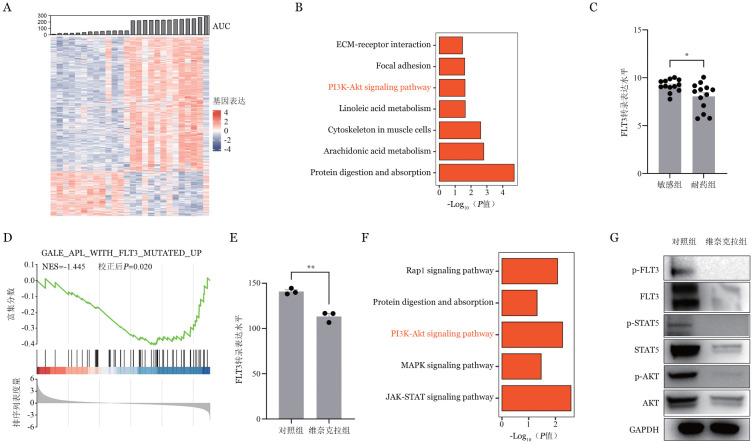
下调差异基因的生物信息学分析及验证（*P<0.05、**P<0.01） **A** 维奈克拉敏感组与耐药组差异基因的热图；**B** 下调基因的KEGG通路富集分析；**C** FLT3在维奈克拉敏感组和耐药组的转录表达水平；**D** 维奈克拉敏感组与耐药组的GSEA富集分析；**E** GSE199159验证队列中FLT3在维奈克拉组和对照组（DMSO）的转录表达水平；**F** GSE199159验证队列中维奈克拉组下调基因的KEGG通路富集分析；**G** Western blot检测维奈克拉组和对照组（DMSO）FLT3信号通路蛋白表达及磷酸化水平

四、耐药基因筛选

耐药组上调基因的KEGG富集分析表明，维奈克拉耐药与转录失调、Toll样受体信号转导、NOD样受体及B细胞受体等信号通路相关（图4A），其中转录失调通路较为显著。为深入了解维奈克拉耐药后转录水平改变，对上调基因进行转录因子富集分析，结果显示TP53、STAT3及FOXM1等转录因子调控网络在耐药组中显著改变（均*P*<0.05）（图4B）。进一步分析发现，耐药组FOXM1、STAT3表达水平显著高于敏感组（均*P*<0.001）（图4C）。为了验证上述发现，在GSE269245数据集中检索相关基因表达水平，结果表明耐药株中FOXM1及STAT3的表达水平显著高于原始株（*P*<0.05）（图4D）。此外，Kaplan-Meier Plotter、TCGA数据库显示，FOXM1或STAT3高表达的AML患者生存时间显著短于低表达组（均*P*<0.05）（图4E~F）。综上，FOXM1、STAT3转录活性升高可能是AML患者对维奈克拉耐药的关键因素，且其表达水平与AML预后密切相关。

**图4:**
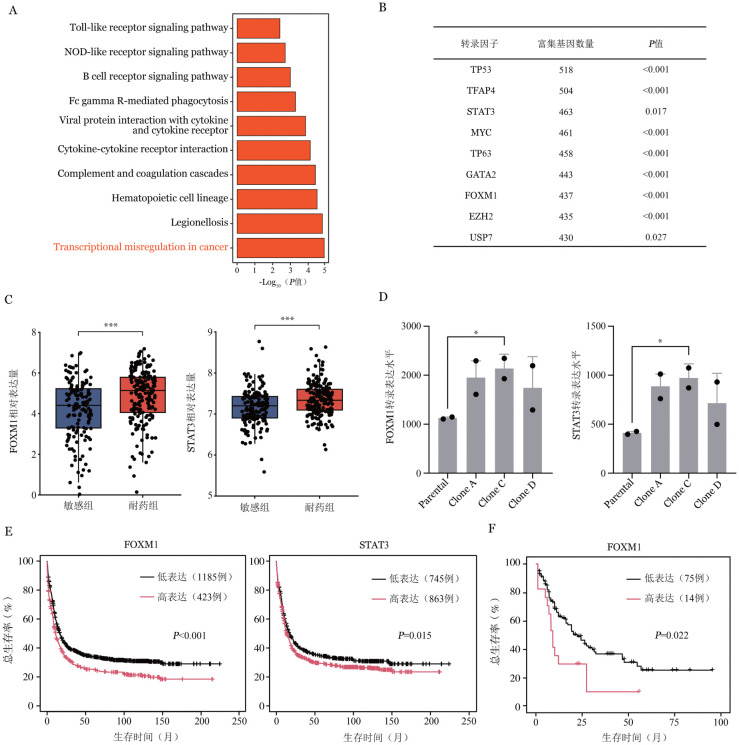
维奈克拉耐药基因筛选及验证（*P<0.05、***P<0.001） **A** 上调基因的KEGG通路富集分析；**B** 上调基因的转录因子富集分析；**C** STAT3及FOXM1在维奈克拉敏感组和耐药组的转录表达水平；**D** GSE269245验证队列中FOXM1及STAT3在维奈克拉耐药株（Clone A/C/D）和原始株（Parental）的转录表达水平；**E、F** 不同FOXM1及STAT3转录表达水平的AML患者的生存曲线

## 讨论

随着各种类型靶向药的应用，AML患者的缓解率已有不同程度的提高[11]，维奈克拉作为最常用的BCL-2抑制剂，显著提高了患者的生存率，但AML患者的高度异质性及维奈克拉的耐药问题仍待解决。因此，探索影响AML患者对维奈克拉治疗敏感性的关键基因及潜在作用靶点至关重要。

现有研究报道，接受维奈克拉联合阿扎胞苷治疗的FLT3-ITD患者属于中度敏感[12]，也存在一定耐药性。本研究我们对Beat AML数据库中AML患者基因突变特征、临床特征、基因表达水平及BCL-2抑制剂维奈克拉药物敏感性等数据进行分析，探索了影响维奈克拉药物敏感性的因素。结果显示，携带FLT3突变（包括FLT3-ITD和FLT3-TKD）的患者对维奈克拉表现出更高的敏感性。但由于FLT3突变（尤其是FLT3-ITD突变）本身会加剧AML的恶性程度，因此，尽管携带FLT3突变的AML患者对维奈克拉敏感，但由于FLT3突变引发的下游信号通路异常高度激活，可能导致AML快速进展[13]，这些患者在接受维奈克拉治疗后，治疗效果与FLT3野生型患者差异无统计学意义。本研究仅探讨了维奈克拉在AML治疗过程中的潜在分子机制，未来研究应进一步细化FLT3突变亚型（FLT3-ITD或TKD）与维奈克拉敏感性的相关性分析，以揭示不同FLT3突变类型对药物反应的具体影响。同时，进一步探索维奈克拉与FLT3抑制剂联用对AML患者的治疗作用。

本研究聚焦于维奈克拉单药治疗的分子机制，通过对维奈克拉敏感和耐药AML患者进行差异基因表达分析及通路富集分析。结果表明，敏感组中的差异基因在PI3K-AKT通路显著富集。PI3K-AKT通路是FLT3下游的关键信号通路之一，能够促进细胞增殖并抑制细胞凋亡[14]–[16]，这提示维奈克拉可能通过抑制PI3K-AKT信号通路在部分患者中发挥抗肿瘤作用。此外，转录组及细胞实验结果也显示，维奈克拉能够抑制FLT3相关信号通路的激活，包括PI3K-AKT、MAPK和JAK-STAT通路，进一步验证了维奈克拉可能通过抑制FLT3信号通路发挥抗AML作用，从而使FLT3-ITD患者从维奈克拉单药治疗中获益。然而，由于FLT3-ITD患者的FLT3信号通路本身已处于异常激活状态[13]，在使用维奈克拉治疗AML的过程中，维奈克拉仅能部分抑制FLT3相关信号通路的活化。因此，FLT3-ITD患者的具体获益及其机制仍需进一步研究。

尽管维奈克拉在临床应用中展现出良好的前景，但耐药性仍是一个重要问题，联合用药策略成为克服维奈克拉耐药的研究热点之一[17]–[18]。已有研究表明，维奈克拉与化疗药物联用能够显著提高AML患者的疗效[19]。化疗药物通过引发细胞DNA损伤促进细胞死亡，而维奈克拉则通过抑制BCL-2的抗凋亡功能，协同增强细胞凋亡。这种联合疗法不仅能克服AML细胞对化疗的耐药性，还能够避免单一治疗策略所带来的耐药问题。本研究发现，维奈克拉耐药患者中上调的基因在转录调控相关通路富集，且与FOXM1、STAT3等转录因子的调控网络改变有关。靶向这些转录因子及其下游信号通路可能是克服肿瘤耐药的有效途径[20]。STAT3在白血病发生过程中起着关键作用，通过调节MYC和SLC1A5的表达，调控氨基酸内流和谷氨酰胺水解等能量代谢途径，进而提高白血病干细胞的氧化磷酸化水平[21]。FOXM1在AML中高表达，是癌症发生和化疗耐药的关键调节因子。在MLL重排的AML中，FOXM1上调激活Wnt/β-catenin信号通路，使白血病干细胞维持静息状态并促进其自我更新[22]，维奈克拉与FOXM1抑制剂NB73联用已被证明能够抑制多发性骨髓瘤的进程[23]。此外，Kaplan-Meier Plotter及TCGA数据库的分析结果也表明，STAT3和FOXM1的高表达与AML患者的不良预后正相关，进一步验证了这两个基因在AML发展中的重要作用。本研究发现，STAT3和FOXM1在耐药组转录水平高于敏感组，FOXM1和STAT3的转录活性升高可能是AML患者对维奈克拉耐药的关键因素。

本研究存在一定局限性：主要基于体外数据，只有52例患者基因组数据、临床信息及转录组数据纳入研究，样本量相对有限。但Beat AML数据库的体外药敏数据已被广泛用于预测临床反应，并在多个研究中得到验证[24]–[26]。然而，AML的耐药机制复杂，仍需进一步开展临床及功能实验，以验证FLT3相关信号通路及FOXM1、STAT3活性对维奈克拉治疗反应性的具体作用机制。

综上所述，本研究通过整合临床数据、转录组数据及基因组数据，探讨了AML靶向BCL-2治疗耐药的潜在分子机制。我们的研究发现，维奈克拉能够抑制FLT3相关信号通路的激活；而STAT3和FOXM1转录因子的活性升高可能是维奈克拉耐药的关键因素。然而，转录因子引发耐药的具体机制仍需进一步验证。总体而言，本研究揭示了STAT3、FOXM1等转录因子网络的改变与维奈克拉耐药的密切关系，靶向这些关键分子有望提高维奈克拉的治疗效果，并缓解耐药问题，为AML的精准治疗提供了新的思路。
